# Lung cancer-associated mesenchymal stem cells promote tumor metastasis and tumorigenesis by induction of epithelial–mesenchymal transition and stem-like reprogram

**DOI:** 10.18632/aging.202732

**Published:** 2021-03-19

**Authors:** Cihui Yan, Jingjing Chang, Xinmiao Song, Ying Qi, Zhenyu Ji, Ting Liu, Wenwen Yu, Feng Wei, Lili Yang, Xiubao Ren

**Affiliations:** 1 Department of Immunology, Tianjin Medical University Cancer Institute and Hospital, National Clinical Research Center of Cancer, Key Laboratory of Cancer Prevention and Therapy, Ti-Yuan-Bei, He Xi 300060, Tianjin, China; 2 Department of Electromyogram, 3rd Affiliated Hospital of Hebei Medical University, Shijiazhuang 050051, Hebei, China

**Keywords:** cancer-associated mesenchymal stem cell, lung cancer, metastasis, epithelial-mesenchymal transition, tumor-initiating cell

## Abstract

Mesenchymal stem cells (MSCs) have attracted more attention in antitumor therapy by using MSCs as vehicles or targeting modulators of MSCs. But their role and mechanisms in tumor progression are less known. In the present study, we successfully isolated pairs of MSCs from lung cancer (LC-MSCs) and adjacent tumor-free tissues. Based on the coculture system *in vitro* and animal studies *in vivo*, we originally found that LC-MSCs significantly promoted tumor metastasis and tumorigenesis both *in vitro* and *in vivo*. Partial epithelial–mesenchymal transition (EMT) was induced in lung cancer cells by LC-MSCs by the evidence of remarkable increase in snail and slug expression but not in other EMT-associated genes. The expression of stem related genes also escalated significantly. And spheroids perfectly formed when tumor cells were co-incubated with LC-MSCs. These results revealed a close link of partial EMT and acquisition of stem-like traits in lung cancer cells which was induced by LC-MSCs and greatly promoted metastasis and tumorigenesis in lung cancer. Our findings provided a new insight into LC-MSCs in tumor progression and helped to identify LC-MSCs as a potential vehicle or target for lung cancer therapy.

## INTRODUCTION

Mesenchymal stem cells (MSCs) are a heterogeneous group of adult progenitor cells, which exist in many tissues. MSCs hold great promise in tissue generation because they are active in homing to the sites of tissue injury where they undergo self-renew and multi-differentiation upon the specific microenvironment [1–3]. Over 700 MSC-based clinical trials are currently listed on the clinical trial registry of the US National Institutes of Health, most of them focused on locally inflammatory control and tissue injuries. There are only four clinical studies that test MSC based therapy in patients with ovarian cancer, lung cancer and neuroblastoma, individually. In these studies, MSCs derived from normal tissues are all used as vehicles delivering antitumor cytokines or cytotoxic agents into patients against cancer. The effect of MSCs on tumors is one of the most important key points determining the safety and outcome of these translational studies.

Tumors are considered to be “wounds that never heal” so it is not surprising that MSCs are recruited to tumor sites by the chemotaxis signals from tumors [4, 5]. MSCs that come from local tissues as well as bone marrow migrate and reside in tumors [6]. Subsequently, they are educated by tumor microenvironment, and then evolve into tumor-associated MSCs (TA-MSCs) or differentiated stromal cells such as cancer-associated fibroblasts (CAFs) [7, 8]. Pericytes in tumors also share some characteristics of MSCs [9, 10]. Several studies in lymphoma [11], hepatoma [12] and breast cancer [13] demonstrated that TA-MSCs or reside MSCs promoted tumor growth and metastasis. There is only one study reporting the lung cancer-associated MSCs (LC-MSCs) as far. And little is known about the association between LC-MSCs and lung cancer. Fully understanding the role of LC-MSCs in progression of lung cancer would benefit more efficient strategies for targeted therapy and using MSCs as vehicles for antitumor treatment.

Epithelial–mesenchymal transition (EMT) is a reversible cellular program that is crucial for embryogenesis, wound healing and malignant progression [14, 15]. In neoplasia, EMT is orchestrated by EMT-inducing transcription factors (EMT-TFs) and play an important role in tumorigenesis, motility and metastasis potential of tumor cells. The characteristics of tumor cells also have a close association with acquisition of stem cell-like properties and high-grade malignancy [16, 17]. Signals from tumor stromal microenvironment are one of the most vital factors that induce EMT [18, 19]. As far, the studies of cytokines that was from tumor microenvironment and induced EMT program, such as TGF-β [20, 21], IL-6 [22, 23] and vascular epidermal growth factor [23], always addressed on CAFs.

TA-MSCs produce large amounts of cytokines. And several of these cytokines have been demonstrated to be associated closely with EMT and tumor metastasis. We therefore hypothesized that LC-MSCs promote metastasis and tumorigenesis by inducting EMT and reprograming stem-like characteristics in lung cancer cells. To test this hypothesis, we cultured primary MSCs derived from pairs of primary lung cancer and adjacent tumor-free tissues and investigated their effect on metastasis and tumorigenesis of the tumor cells, addressing EMT and stem-like characteristics.

## RESULTS

### Characteristics of MSCs derived from primary lung cancer tissues

**Figure 1:**
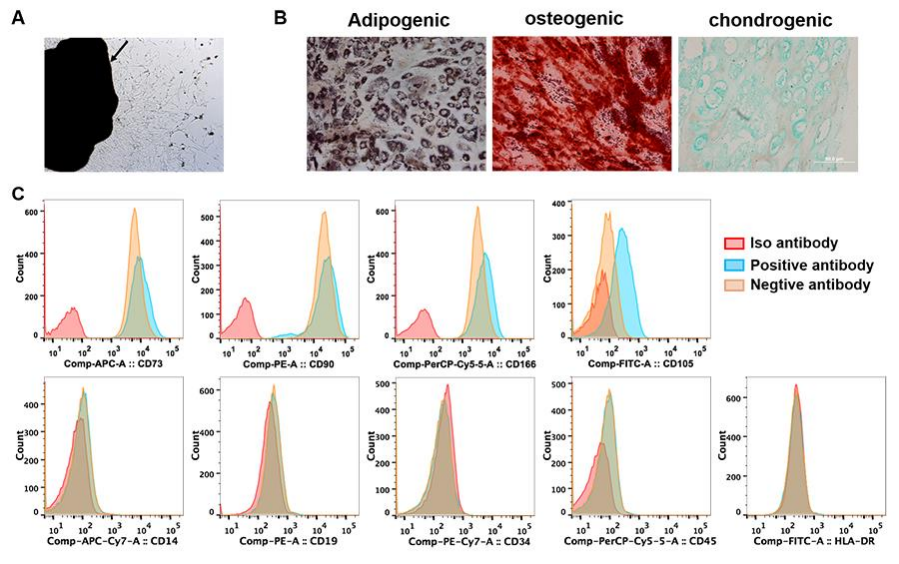
**Identification of lung cancer-associated mesenchymal stem cells.** (**A**) Tissue block culturing method was used to isolate MSCs from matched primary lung cancer and adjacent tumor-free tissues. Arrow, lung cancer tissue. (**B**) Adipogenic, osteogenic and chondrogenic differentiation was induced in MSCs *in vitro*. (**C**) The expression of surface markers of MSCs was tested by cytometry flow. (**A**–**C**) were representative results from LC-MSCs of one patient. LC-MSCs, lung cancer-associated mesenchymal stem cells.

We firstly identified the cells isolated from primary tumor and tumor-free tissues. A few of adherent cells crawled out from both tumor and tumor-free tissues of all patients after 5 days of initial plating. Multiple fibroblast-like cells distributed spirally around or beside the tissue blocks two weeks later (Figure 1A). Flow cytometry analysis showed the cells isolated from tumor tissues were all positive for CD73, CD90 and CD166, and negative for hematopoietic markers of CD14, CD19, CD34, CD45 and HLA-DR. 55-85% cells expressed CD105 (Figure 1C). When cultured in adipogenic, osteogenic or chondrogenic induction medium, these cells differentiated into adipose cells, osteocytes and chondrocytes, individually (Figure 1B). The cells isolated from tumor or tumor-free tissues showed no significant difference in morphology, phenotype and multidifferential potency except that the percentage of CD105 expression was in a wider range (15%-80%) in the cells derived tumor-free tissues. These results indicated that we isolated MSCs form primary lung cancer (LC-MSCs) and adjacent tumor-free tissues (TF-MSCs) successfully.

### Increased invasion in tumor cells by LC-MSCs *in vitro*

**Figure 2:**
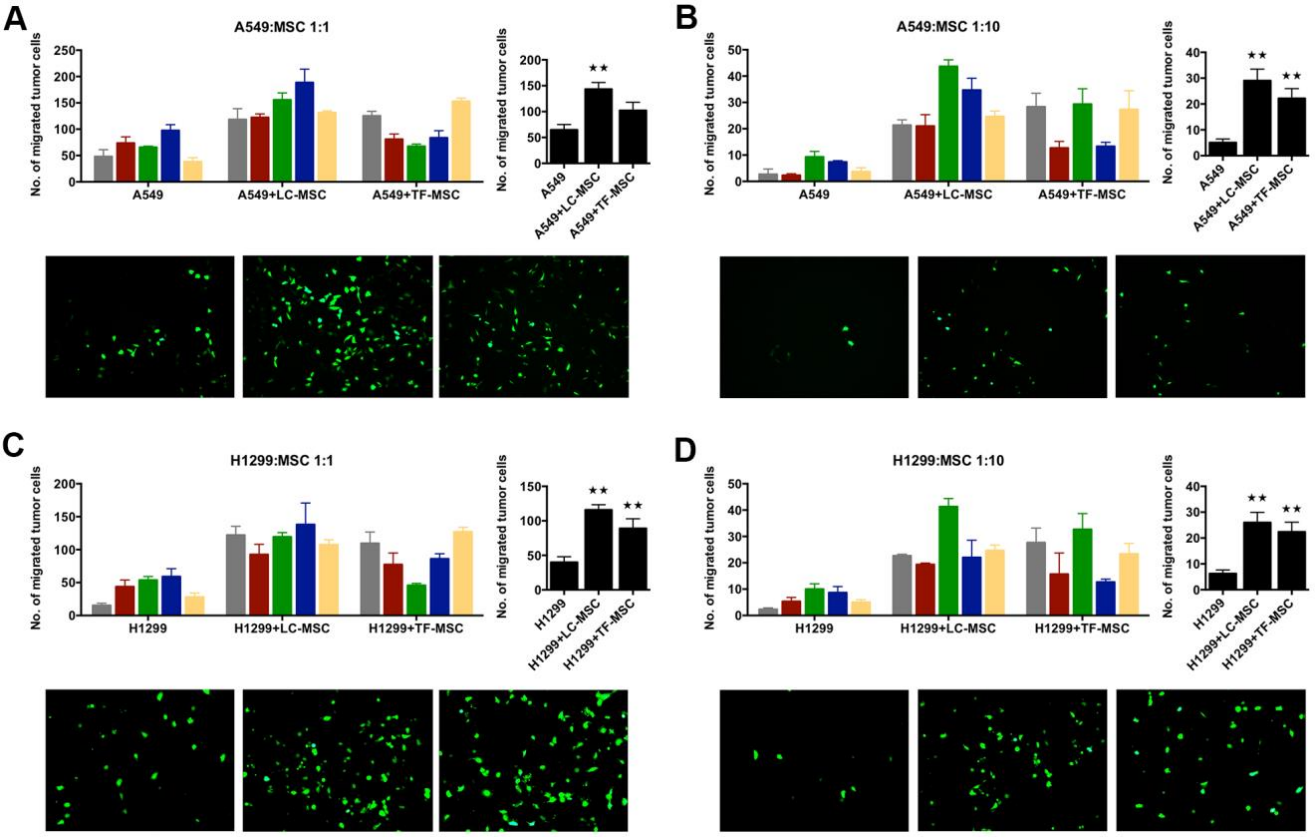
**Tumor invasion was induced by LC-MSCs *in vitro*.** A549.CopGFP and H1299.CopGFP cells were transduced by lentiviral particles to express CopGFP stably firstly. Then the invasion ability of these cells was tested by transwell invasion assay after they were co-incubated with LC-MSCs or not for 24 hours. (**A**, **B**) A549 cells were co-incubated with LC-MSCs at the cell ratio 1:1 and 1:10. (**C**, **D**) H1299 cells were co-incubated with LC-MSCs at the cell ratio 1:1 and 1:10. Color bar chart, triplicate data for each patient. Dark bar chart, statistical analysis for all patients. Fluorescent pictures illustrated tumor cells that had migrated through the membrane of transwell inserts. TF-MSCs, tumor-free mesenchymal stem cells, were used as comparison from normal lung tissues. **, P < 0.05 were considered to be statistically significant.

To investigate how LC-MSCs influenced lung cancer cells, we co-cultured tumor cells with LC-MSCs *in vitro* and examined the invasion of tumor cells firstly. A549 and H1299 cells expressed CopGFP fluorescence stably by lentivirus transduction. Transwell assay showed the number of migrated tumor cells dramatically increased to twice to six times when tumor cells were co-cultured with LC-MSCs at the cell ratio of 1:1 as well as 1:10 (Figure 2A–2D). We also co-cultured tumor cells with TF-MSCs. Although TF-MSCs also increased tumor invasion, they were not as effective as LC-MSCs (Figure 2A–2D). Additionally, the supernatants of LC-MSC showed similar effect on migration in tumor cells (Supplementary Figure 1). These results indicated that LC-MSCs increased the invasion ability of tumor cells.

### Changes of migration in tumor cells by LC-MSCs *in vitro*

**Figure 3:**
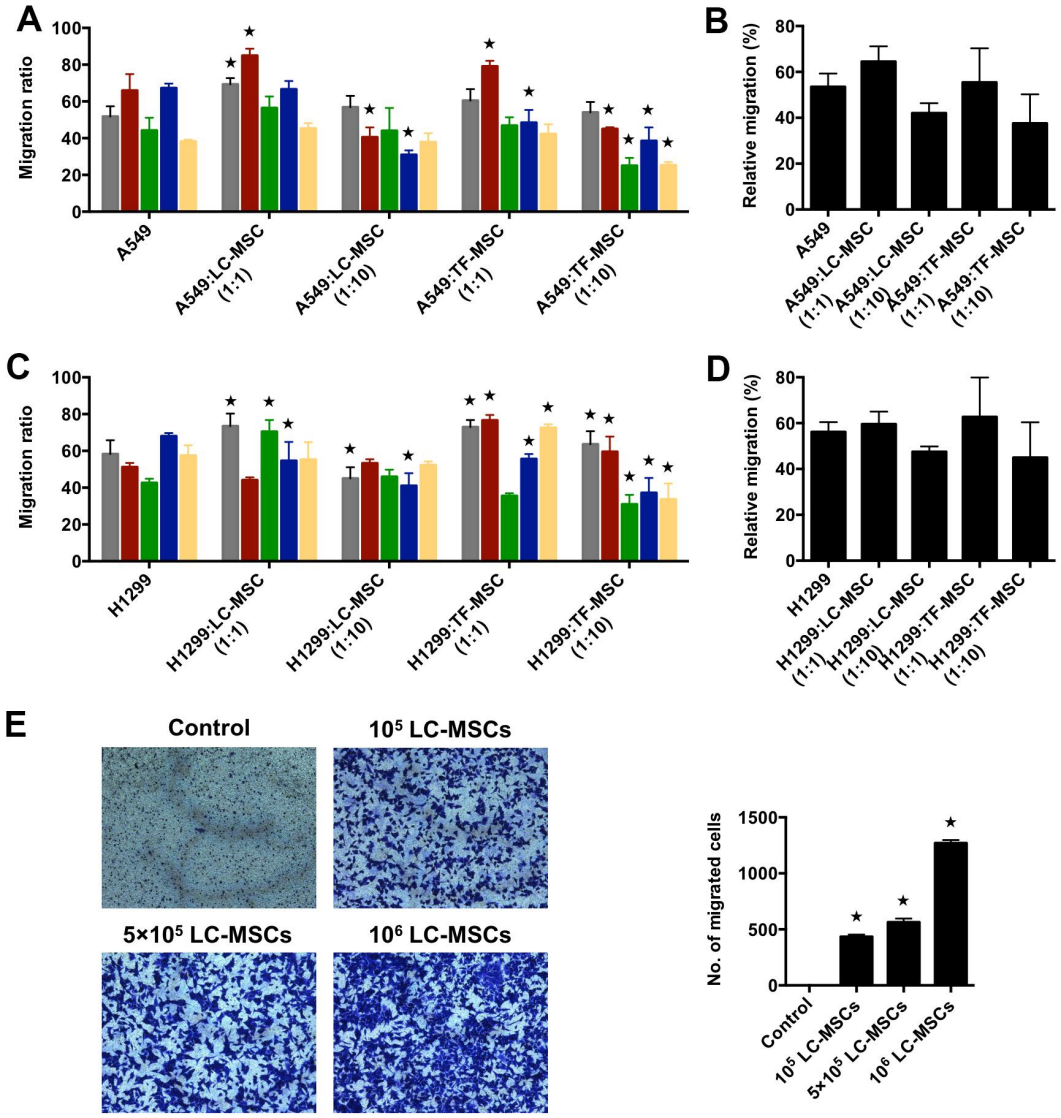
**Tumor migration influenced by LC-MSCs *in vitro*.** (**A**–**D**) A549.CopGFP and H1299.CopGFP cells were co-incubated with LC-MSCs at the cell ratio 1:1 and 1:10 to 80% confluence before wound healing assay was performed. The migrated ratio was calculated as (the first scratch distance - the scratch distance 48 hours later)/ the first scratch distance×100%. Color bar chart, triplicate data for each patient. Dark bar chart, statistical analysis for all patients. TF-MSCs, comparison from normal lung tissues. (**E**) The migratory capacity of A549 cells in response to conditioned medium of LC-MSCs was determined using transwell migration assay. Control, serum-free medium without LC/TF-MSCs. *, P < 0.05 were considered to be statistically significant.

Next, we used scratch wound healing assay to evaluate the migration ability of tumor cells in co-culture system. We firstly analyzed the results according to each patient. As shown in Figure 3A, 3C, at the cell ratio of 1:1, the migration rate increased significantly in three patient-derived LC-MSC co-culture systems and decreased in one system when compared with that when tumor cells were cultured alone. While, at the cell ratio of 1:10, it did not elevate and even ran down to statistic differences in three co-culture systems (Figure 3A, 3C). TF-MSCs had a similar effect on the migration of tumor cells as LC-MSCs (Figure 3A, 3C). Secondly, we analyzed the migration rate between co-culture and tumor culture alone system by pooling all the data from individual patients, but did not find significant differences (Figure 3B, 3D). Then, we used transwell assay to test the migration of tumor cells to LC-MSC-derived condition medium. The result revealed a great many of tumor cells migrated to the condition medium of LC-MSCs in a concentration-dependent manner. Contrastively, few tumor cells migrated to serum-free medium (Figure 3E). These results suggested tumor cells had tropism to LC-MSCs.

### Induced EMT-associated gene expression in tumor cells by LC-MSCs

**Figure 4:**
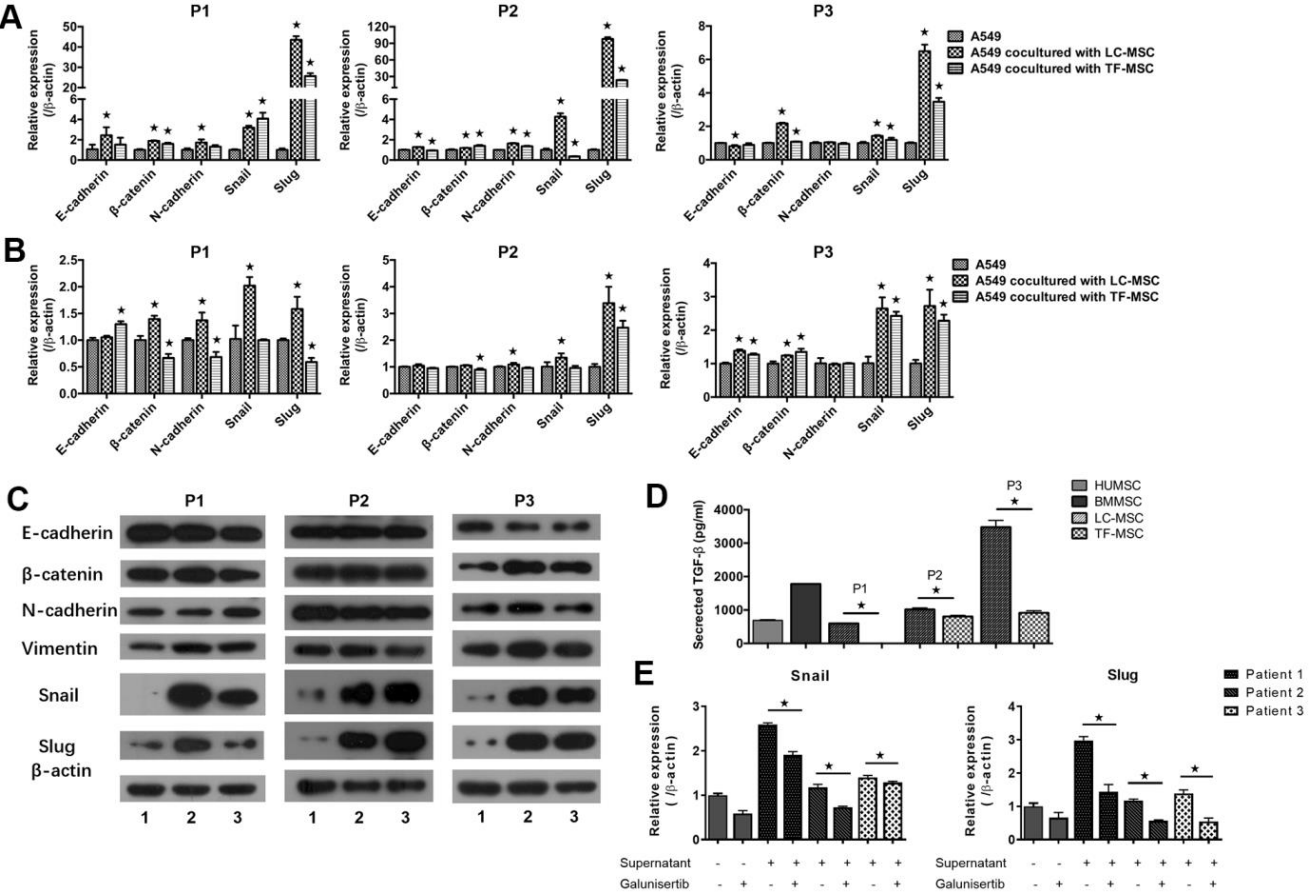
**EMT-associated gene expression induced by LC-MSCs.** (**A**) Equal number of A549.CopGFP and LC-MSCs were co-cultured directly for 48 hours and then sorted by cytometry flow to test the expression EMT-associated genes by realtime PCR. (**B**, **C**) Equal number of A549.CopGFP and LC-MSCs were co-incubated separately by transwell inserts for 48 hours. The EMT-associated gene expression in A549 cells was evaluated by realtime PCR (**B**) and western blot (**C**), individually. Representative results of three patients. TF-MSCs, comparison from normal lung tissues. (**D**), The level of TGF-β secreted by MSCs that derived from different tissues was tested by ELISA assay. (**E**), The induced snail and slug expression was blocked by TGF-β signaling inhibitor galunisertib. A549 cells were pre-incubated with galunisertib (5 μM) for 30 min, then treated by the supernatants of LC/NC-MSCs from 3 patients. *, P < 0.05 were considered to be statistically significant. P1-3, three patients. 1, Tumor cells alone. 2, Tumor cells + LC-MSCs. 2, Tumor cells + TF-MSCs. Data from triplicate experiments.

**Figure 5:**
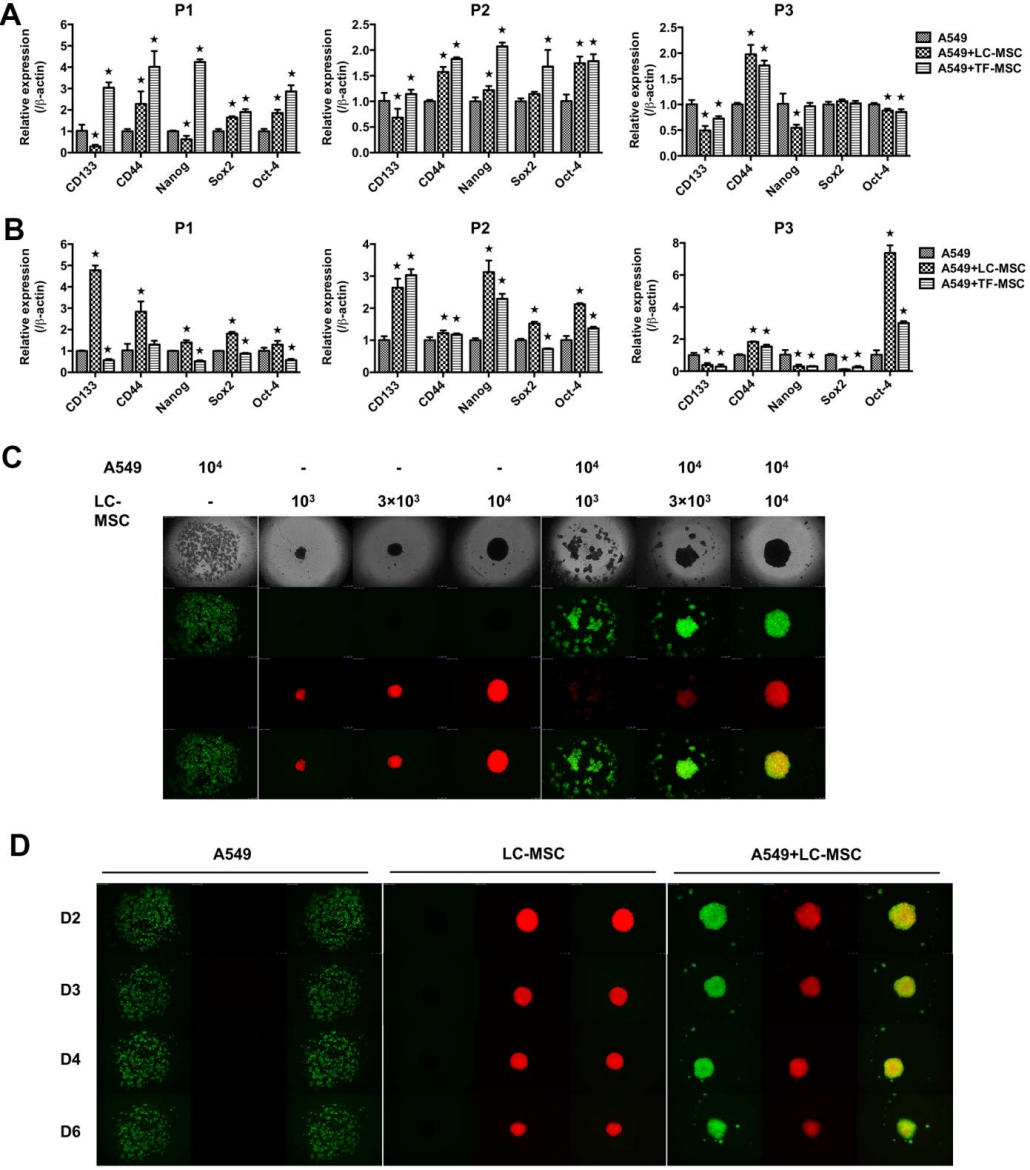
**Stem-like characteristics induced by LC-MSCs.** (**A**) Equal number of A549.CopGFP and LC-MSCs were co-cultured directly for 48 hours and then sorted by cytometry flow to test the stem-related gene expression by realtime PCR. (**B**) Equal number of A549.CopGFP and LC-MSCs were co-incubated separately by transwell inserts for 48 hours. The expression of stem-related genes in A549 cells was evaluated by realtime PCR. (**C**) Different cell ratio of A549.CopGFP and LC-MSCs. DsRed were co-incubated in 96-well 3D cell culture plates. The spheroid formation was observed under inverted fluorescence microscope on the second day of plating cells. (**D**) Spheroid grew observed at indicated time. *, P < 0.05 were considered to be statistically significant. P1-3, three patients. Data from triplicate experiments.

To investigate the mechanism of enhanced invasion of lung cancer cells, we then tested the expression of EMT-associated genes in transcriptional as well as protein levels. A549 cells that expressed CopGFP florescence were firstly sorted from the co-culture systems by cytometry flow. Realtime PCR analysis showed the expression of EMT-associated transcription factors snail and slug elevated remarkably in A549 cells when co-cultured with LC-MSCs derived from all patients and the change of slug expression was the most significant (snail, 1.4 to 4.3 times; slug, 6.5 to 98 times) (Figure 4A). E-cadherin, N-cadherin and β-catenin expression also change obviously in the co-culture systems (Figure 4A). Furthermore, to determine whether LC-MSC-induced EMT in the tumor cells was cell-contact dependence, we cultured tumor cells and LC-MSCs separately by using transwell inserts. A same trend but relative slight changes of the EMT-associated gene expression was found in this indirectly contacted condition (Figure 4B). Western blot results confirmed ascent expression of snail and slug in A549 cells in co-culture system (Figure 4C). Vimentin expression increased. But E-cadherin, β-catenin and N-cadherin did not exhibit much changes in A549 cells (Figure 4C). TF-MSCs increased the EMT-associated gene expression as well but in a lower level than LC-MSCs (Figure 4A–4C). Additionally, we found much higher level of TGF-β secreted from LC-MSCs compared with that from TF-MSCs (Figure 4D). And blockade of TGF-β downstream signaling by galunisertib decreased the levels of snail and slug significantly in A549 cells (Figure 5E). These results indicated that TF-MSCs might promote EMT by TGF-β signaling pathway.

### Induced stem-like characteristics of tumor cells by LC-MSCs

As EMT program have a close association with acquisition of stem-like traits in tumor cells, we next assessed the stem-like characteristics in tumor cells in both cell-cell contact (Figure 5A) and noncontact culture condition (Figure 5B). When tumor cells were co-cultured with LC-MSCs directly, we found the level of CD44 but not CD133 in tumor cells increased in all 3 patients (Figure 5A). Enhanced expression of Oct-4 with either Sox2 or Nanog was found in patient 1 and 2. While none of them increase in patient 3 (Figure 5A). LC-MSCs did not show superior capacity to promote stem gene expression in tumor cells compared with TF-MSCs in the cell-cell direct contact system (Figure 5A). However, all the tested genes increased when tumor cells were incubated with LC-MSCs by transwell insert in patient 1 and 2 (Figure 5B). CD44 and Oct-4 increased in patient 3 (Figure 5B). And the level of stem genes increased more significantly induced by LC-MSCs compared with that by TF-MSCs in the cell-cell indirect culture system (Figure 5B). Furthermore, we tested spheroid formation in 3D cell culture system. A549 cells and MSCs were labeled with CopGFP and DsRed florescence, individually. As shown in Figure 5C, on the second day after plating, A549 cells tiled loosely in the bottom of cell culture well where they were plated alone. On the contrary, the cells aggregated in the wells in co-culture condition. The degree of compaction depended on the absolute cell number of LC-MSCs and its ratio to A549 cells plated in each well. The most perfect spheroids were found when cells were co-incubated at the ratio of 1:1. With the time of culturing, the spheroids turned more compacted. MSCs accumulated in the center of the spheroid and A549 cells located in the outer part of the spheroid (Figure 5D). Spheroid also formed when A549 cells were co-incubated with TF-MSCs. But they were not as compacted and round as that when A549 cells were co-incubated with LC-MSCs (Supplementary Figure 2).

### Enhanced tumor growth and metastasis by LC-MSCs *in vivo*

**Figure 6:**
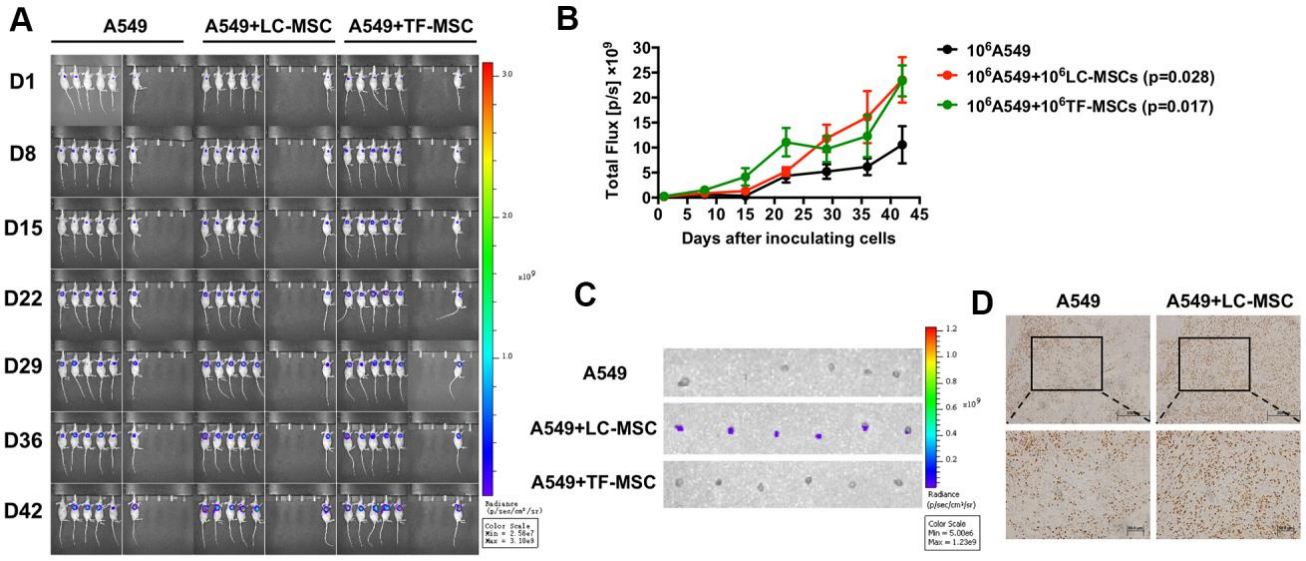
**Tumor growth and metastasis promoted by LC-MSCs.** 10^6^ each of A549.Luc cells and LC-MSCs or TF-MSCs were co-implanted subcutaneously into female Balb/c nude mice. Tumor growth was monitored by bioluminescence imaging. (**A**) Bioluminescence images at the indicated time. (**B**) Total bioluminescence of tumors at indicated time. (**C**) Bioluminescence images of sentinel lymph nodes from sacrificed mice at the end of experiment. (**D**) Immunohistochemistry test for Ki67 in tumor tissues. *, P < 0.05 were considered to be statistically significant.

Subsequently, we examined the effect of LC-MSCs on tumor progression *in vivo* by live animal imaging. 10^6^ each of A549.Lu cells and LC/TF-MSCs were co-implanted subcutaneously in mice. The BLI revealed that tumor grew faster in both A549+LC-MSC and A549+TF-MSC group than A549 group. And there was no significant difference of tumor size between the two co-implanted groups (Figure 6A, 6B). Tumors in A549 group gradually grew slowly at the late feed period. When dissecting the sacrificed mice at the end of study, we found the tumor parenchyma was usually occupied by cystic cavities in A549 group, but it was not found in A549+LC-MSC group. H.E staining indicated that the tumor cells in the co-implanted groups presented more derangement distribution which was disordered by fibroblast-like cells than that in A549 group, (Supplementary Figure 3). Interestingly, bioluminescence signaling was detected in sentinel lymph nodes in all mice in A549+LC-MSC group, but not in the other two groups (Figure 6C). Immunohistochemistry result showed a higher level of ki67 staining in the co-implanted group (Figure 6D).

### Increased tumorigenesis and metastasis-initiating ability by LC-MSCs *in vivo*

**Figure 7:**
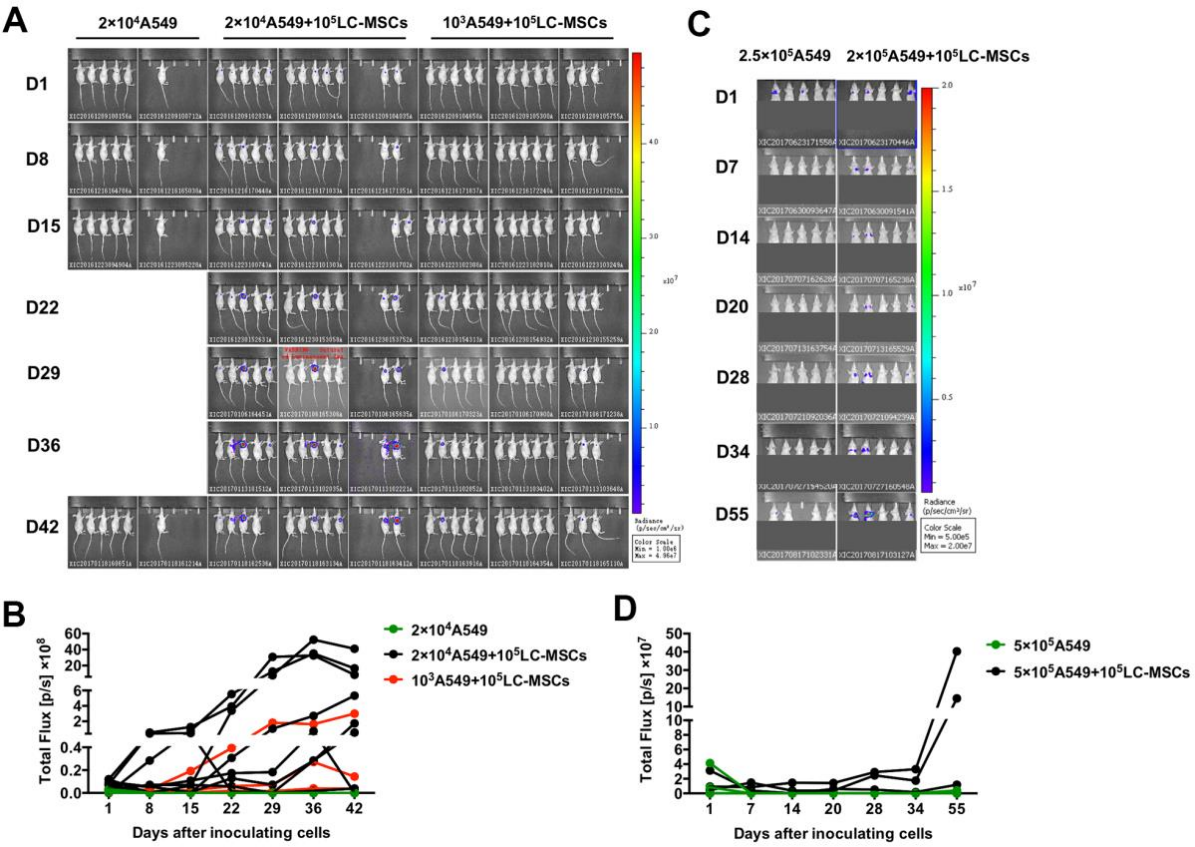
**Increased tumorigenesis and metastasis-initiating ability by LC-MSCs.** (**A**, **B**) Low number of A549.Luc cells and LC-MSCs were co-implanted subcutaneously into female Balb/c nude mice at different cell ratio. Bioluminescence images (**A**) and total bioluminescence of tumors (**B**) at the indicated time. (**C**, **D**) A549.Luc cells and LC-MSCs were mixed well and injected intravenously into female Balb/c nude mice. Bioluminescence images (**A**) and total bioluminescence of tumors (**D**) at the indicated time.

As LC-MSCs induced stem-like characteristics in tumor cells, we decreased the number of implanted tumor cells to investigate the pro-tumorigenesis of LC-MSCs *in vivo*. As shown in Figure 7A, 7B, tumor grew in seven out of twelve and three out of thirteen mice when 2×10^4^ or 10^3^ A549 cells were co-implanted with LC-MSCs. However, no tumor developed when 2×10^4^ A549 cells were implanted alone. We also tested the effect of LC-MSCs that derived from another patient and got the similar result (Supplementary Figure 4 and Table 1). Furthermore, we investigated whether LC-MSCs promoted the metastasis-initiating ability and colonization of circulating tumor cells by intravenous injection of tumor cells in mice model. The result revealed that three out of five mice in A549+LC-MSC group developed lung cancer. Contrastively, there was only one mouse developed lung cancer in A549 group. Meanwhile, the bioluminescent signaling from it was much weaker than that in A549+LC-MSC group and just detected in the last imaging (Figure 7C, 7D).

**Table 1:** Limiting dilution analyses of tumorigenic lung cancer cells.

**Co-implanted cells**	**no. tumors/ no. injections**		**Lung cancer-initiating cell frequency (95% CI) (‰)**		
	**cells/injection**				
	**20,000**	**1,000**	**Estimate**	**Lower**	**Upper**
**A549+LC-MSCs-P1**	**7**/12	**3**/13	**0.117**	0.048	0.284
**A549+LC-MSCs-P2**	**5**/7	**4**/7	**0.059**	0.030	0.118
**A549**	0/7, 0/6	0/7	-	-	0.011

A549.Luc cells were implanted into female Balb/c nude mice with LC-MSCs or not. The frequency of lung cancer-initiating cells was calculated by limiting dilution analyses (http://bioinf.wehi.edu.au/software/elda/index.html). In all cases, lung cancer-initiating cells were significantly (p<0.05) more frequent in the co-implanted groups. CI: confidence interval.

## DISCUSSION

In the present study, we successfully identified MSCs from primary lung cancer as well as tumor-free adjacent tissues. These cells promoted lung cancer tumorigenesis and metastasis both *in vivo* and *in vitro*. Our study provided the first evidence that EMT and reprogram of stem-like traits which were induced by lung cancer-associated MSCs were involved in the tumor progression.

Enzyme digestion and tissue block culturing are the two major methods that have been widely used to isolate MSCs from adult tissues. In the present study, we used tissue block culturing method. As such, the cells crawled out from the tissues themselves without external influence, which could avoid multiple cell types were mixed in the primary and earlier culture cells. Meanwhile, no additional grow factors except FSC were added in culture medium, which was hard to support the non-MSC cells such as tumor cells, fibroblasts and endothelial cells to grow because they were more urgent for specific growth factors and cytokines to grow *in vitro*. As a result, we acquired much purer MSCs from the first passage with a success rate of 100%. The isolated MSCs had unique morphology, biomarker and multi-differentiation potential as previous studies reported except that they heterogeneously expressed CD105. Similar to our result, the mice adipose-derived MSCs had CD105^+^ and CD105^-^ subsets, and CD105^-^ MSCs had improved differential ability and enhanced efficiency to inhibit the proliferation of CD4^+^ T cells compared with CD105^+^ MSCs [24]. Other studies showed the human peritoneal dialysis effluent-derived and skin-derived MSCs were negative for CD105 expression [25, 26]; and the lack of CD105 did not influence the multi-differentiation of these cells [25, 27]. CD105 expression is affected by culture time, cell confluence and different derivation resources. It is probable that the specific tumor environment of lung cancer educates the heterogeneous expression of CD105 on tumor-derived MSCs, which might benefit tumor progression.

Next, we found that LC-MSCs promoted invasion and migration of tumor cells *in vitro*. Tumor cells showed mesenchymal-like morphologic change in co-incubated systems. We used two kinds of cell ratios, tumor cells versus LC-MSCs 1:1 and 1:10, in co-culture system in order to mimic the interaction between tumor cells and MSCs at the border of tumor and tumor metastasis where tumor cells were surrounded by stromal cells, respectively. The improved invasion was more significant at cell ratio of 1:10, which indicated LC-MSCs played a vital role in invasion change. This result was supported by previous reports that TA-MSCs produced pro-metastatic factors such as chemokines to promote tumor cell invasion and metastasis by acting on tumor cells as well as TA-MSCs [28, 29]. Mice bone marrow-derived MSCs that secreted CC-chemokine ligand 5 (CCL5) promoted tumor cell motility, invasion and metastasis by CCL5/CCR5 signaling pathway [7]. But it was not the case in our study because both the tumor cells and MSCs did not or expressed very low level of CCR5 (Supplementary Figure 5). Interestingly, we found LC-MSCs produced multiple kinds of matrix metalloproteinases (MMPs) in much higher levels in comparison with human bone-marrow derived MSCs, which could break down the extracellular matrix, and then facilitate tumor migration (the data forms part of an ongoing study). This might be one of the reasons resulting in enhanced invasion of tumor cells by LC-MSCs in transwell assay. However, we did not find enhanced tumor migration in wound scratch assay. And it tended to decrease when tumor cells were co-incubated with LC-MSCs at a lower ratio (1:10). It was probably because there was closely mutual attraction between these LC-MSC and tumor cells by the evidence of tumor tropism to LC-MSCs (Figure 3E) and compacted spheroids formed in 3D culture (Figure 5C, 5E) in our present study as well as our previous results that MSCs were inherently prone to cancer cells, such as lung cancer cells [30] and hepatocarcinoma [31]. When tumor cells were incubated with LC-MSCs in even culture media at low cell number ratio, mutual attraction played a much more important role among these cells. As a result, the tumors cells preferred intensive interaction with LC-MSCs but not move toward the wound scratch.

Although we did not find LC-MSCs influenced the growth of tumor cells *in vitro* (data not shown), the results from animal study revealed that LC-MSCs facilitated not only the tumor growth but also invasion *in vivo*. Firstly, we excluded the tumorigenic potential of MSCs themselves as we found no tumor formed when LC-MSCs or TF-MSCs were implanted alone in mice. Our result was consistent with the studies that bone marrow-derived MSCs (BM-MSCs) promoted colorectal cancer progression through paracrine neuregulin 1/HER3 signalling [32] and tumor formation of multiple myeloma by cytokine IL-6 which was delivered via MSC-derived exosomes [33]. Additionally, BM-MSCs also contributed to tumor angiogenesis by secreting pro-angiogenic factors, such as VEGF [34] and IL-6 [35], as well as to differentiating into pericyte-like cells [36, 37]. These studies also supported our finding that tumors gradually lost their expansion ability at the late feed period in A549 group, while still exhibited vigorous growth power in the co-implanted group. Furthermore, we found lymph node metastasis only occurred in A549 + LC-MSCs group (5/5). These interesting findings promoted us explore the underlying mechanism involved in.

We found EMT in A549 and H1299 (Supplementary Figure 6) cells was greatly induced by LC-MSCs. The expression of EMT-TFs snail and slug in tumor cells increased significantly in co-incubated system. Similar to our results, a report demonstrated that expression of snail played an essential role in the dissemination of mouse carcinoma cells [38], and another study revealed that expression of slug in primary mammary epithelial cells greatly increased their metastatic capacity [39]. Although there is no direct evidence that TA-MSCs induce EMT in tumor cells as far, accumulated studies have shown CAFs which are an important component of the tumor environment and might derived from MSCs [40, 41] are capable of inducing EMT in tumor cells by paracrine signaling pathways. Consistent with several previous studies [20, 21], our finding indicated that TGF-β secreted from LM-MSCs might play an important role in inducing EMT in tumor cells. Moreover, we found the levels of EMT-TFs in tumor cells escalated even more remarkable in direct co-cultures system compared with that in condition media from LC-MSCs. The indirect effects were mediated by paracrine cytokines as well as secretome or the EVs from MSCs. However, when LC-MSCs and tumor cells were mixed together, membrane-membrane contact and thus signaling transduction also involved and might play more important role in LC-MSC-induced EMT in lung cancer. Meanwhile, the EMT program induced by LC-MSCs was in a partial state by the evidence of medium to slight changes of expression of E-cadherin, β-catenin, N-cadherin and vimentin. Partial or intermediate EMT has been defined as the coexpression of epithelial and mesenchymal traits. Multiple EMT-TFs, their downstream genes and other associated factors have their respective characteristics when cells respond to external stimulation, and orchestrated and activate EMT. It is not necessary and impossible that all of them exhibit consistent and great changes in EMT process [42, 43]. Actually, complete EMT rarely occurs during the progression of human carcinoma [42, 43]. A recent study reported single-cell RNA sequencing to profile mammary epithelial cells undergoing a spontaneous spatially determined EMT. Pseudospatial trajectory analysis identified continuous waves of gene regulation. And cells positive for both E-cadherin and vimentin were the most frequent in the second half of the trajectory [44]. Nerveless, our results suggested that partial EMT induced by LC-MSCs resulted in improved invasion and metastasis in lung cancer cells. Further study need emphasize the transient and dynamic gene expression during LC-MSC induced partial EMT.

Additionally, LC-MSCs facilitated the tumor cells to acquire stem-like characteristics by the evidence of escalated expression of stem associated genes, spheroid formation in 3D culture and tumorigenesis *in vivo*. LC-MSCs were prone to form spheroids because of their stem characteristics. A549 cells always kept a dispersion state as extreme low level of stem-initiating cells existed (Table 1). As a result, it was probably that the reciprocal attraction between tumor cells and LC-MSCs, and the most important, the effect of LC-MSCs on tumor cells led to formation of spheroids in co-culture system. Actually, the clustering tendency of these two types of cells was found just after 2 hours of co-incubation. And with the time went on, spheroids became more compacted and exhibited the characteristic distribution that LC-MSCs located at the center and tumor cells surrounded them. This natural distribution might be optimal to the mutual contact among these cells for signal transmission besides paracrine mechanism. Mutual contact might be more benefit tumor cells to acquire stem-like trait as the expression levels of stem-related genes increased even much higher in direct culture condition than that in transwell insert culture. This was not the same with the changes of EMT-associated genes that the expression of slug and snail escalated higher in transwell insert culture. These results suggested that both mutual contact interaction and paracrine regulation contributed to induction of EMT as well as stem-like reprogram although they might affect in different focus. Consistent with our result, several studies also documented a close link between EMT program and the acquisition of stem-like characteristics in colon, breast, lung and pancreatic carcinomas [45, 46]. Interestingly, we found partial EMT and acquisition of stem-like traits simultaneously existed in the co-incubated system. A similar observation also showed EMT generated CSCs, which appear to reside at an intermediate state along the epithelial–mesenchymal spectrum [47]. EMT-TFs snail and slug that showed increased expression probably acted as a pivot hub involving in EMT program and CSCs arising in tumor cells [45, 46].

We used TF-MSCs as corresponding normal control in the study. The normal lung tissues collected were all more than 5 centimeters away from tumor tissues to exclude the underlying tumor metastasis. The specific characteristics that distinguished tumor-derived MSCs from its corresponding tumor free-derived MSCs were little known. We found here LC-MSCs and TF-MSCs had similar effect on tumor proliferation *in vivo*, but their impact on lymph node metastasis was dramatically different. The evidence of stronger ability of LC-MSCs to increase tumor migration and EMT compared with TF-MSCs *in vitro* confirmed the result *in vivo*. Interestingly, we found divergent levels of stem-like genes in tumor cells induced by TF-MSCs in cell-cell direct and indirect contact culture system, which indicated additional mechanism resulted from cell-cell interaction. However, TF-MSCs failed to help tumor cells to form 3D spheres as compact as LC-MSCs did. All these results demonstrated that LC-MSCs was superior to TF-MSCs in facilitating tumor metastasis, and the effect of LC-MSCs was strengthened *in vivo*.

Finally, genetically modified MSCs delivering cytotoxic genes and targeting upstream and downstream modulators of TA-MSCs have shown two promising strategies for targeted tumor therapy in basic studies. But there is still a long way to safely and effectively translate them into clinical before comprehensive understanding the role of MSCs in tumor progression. For the first time, our results revealed that MSCs residing in lung cancer tissues facilitated the tumorigenesis and invasion. However, as TA-MSCs possess an altered secretome of growth factors depending on their different derivation, progressive stages and tumor environment [48], the detail mechanisms of destiny of TA-MSCs and their reciprocal interaction with tumor cells is needed further study. Additionally, TF-MSCs had much weaker effect on tumor invasion and metastasis compared with LC-MSCs although TF-MSCs and LC-MSCs exhibited similar ability to promote tumor growth. Deep comparison of these cells from different sources would provide novel clues in MSC study.

## CONCLUSIONS

This study original demonstrated LC-MSCs promoted the tumorigenesis and metastasis of lung cancer by inducing epithelial-mesenchymal and stem-like reprogram, which developed new insight to design optimal strategies for MSC-based therapy against tumor. The in-depth mechanism by which LC-MSCs deteriorate tumor progression is unclear and warrants further investigation.

## MATERIALS AND METHODS

### Ethics approval and consent to participate

The informed consent documents were approved by the participating local institution’s review boards, and all patients were informed in the study which was undertaken in accordance with Declaration of Helsinki. Animal research was performed in compliance with the Guide for the Care and Use of Animal Ethics Committee of Tianjin Medical University (Tianjin, PR China).

### MSCs preparation

MSCs were isolated from five pairs of matched primary lung cancer and adjacent tumor-free tissues from the patients (Supplementary Table 1) who newly diagnosed as lung cancer and received lung surgery in Tianjin Medical University Cancer Institute and Hospital (Tianjin, China). Tissue culture method was used to isolate MSCs. Briefly, after lung surgical resection, tissues were immersed immediately in phosphate-buffered saline (PBS) supplemented with 100 units/mL penicillin and 100 μg/mL streptomycin (Gibco, USA) at 4° C till use within 4 hours. Tissues were rinsed with ice-cold PBS to remove the blood and cut into blocks with the size of 8 mm^3^ with scalpels. During cutting, remove the vessels completely. Tissue blocks were plated on the T-75cm^2^ cell culture flasks (Corning, USA) which had been moistened with 2 mL culture medium including DF-12 (Gibco, USA) supplemented with 2 mmol/L L-glutamine (Gibco, USA) and 10% FSC (Gibco, USA) and incubated upside down at 37° C in a humidified atmosphere containing 5% CO_2_ overnight. The flasks were turned over inversely in the second morning. 1 mL culture medium was added into the flasks for the first three days continually and 3 mL culture medium was added on the fourth day. Then culture medium was changed half every three days. MSCs were passaged firstly after two weeks of plating. Cells were subcultured at a density of 4000 cells/cm^2^ in culture medium. MSCs at passages 1 to 3 were cryopreserved with cryprotectant consisted of 45% DF-12, 50% FCS and 5% DMSO (Gibco, USA) in liquid nitrogen for long term storage. MSCs at passages 4 to 6 were used for the following experiments.

### Cell line culture

The human lung cancer cell line A549 and H1299 (bought from Cell Resource Center of Shanghai Institute of Life Sciences, Chinese Academy of Sciences, Shanghai, China) were cultured in RPIM1640 (Gibco, USA) supplemented with 2 mmol/L L-glutamine, 100 units/mL penicillin, 100 ug/mL streptomycin and 10% FCS. The human embryonic kidney cell derived 293 T cell line (kindly provided by Professor Dongsheng Xiong, Institute of Hematology and Blood Diseases Hospital, Chinese Academy of Medical Sciences and Peking Union Medical College, PUMC, Tianjin, China) was maintained in DMEM (Gibco, USA) supplemented with 2 mmol/L L-glutamine, 100 units/mL penicillin, 100 ug/mL streptomycin and 10% FCS. Cells were incubated at 37° C in a humidified atmosphere containing 5% CO_2_.

### Cell phenotype identification

To identify the phenotype of isolated cells from primary lung cancer and adjacent tumor-free tissues, flow cytometry was used to detect the surface markers on these cells. When growing to the confluence of 70%, cells were enzymed and rinsed with ice-cold PBS twice. Cells were suspended at the density of 10^7^/mL with staining buffer and aliquoted to 100 μL. Fluorescein-conjugated mouse anti-human CD73, CD90, CD105, CD166, CD14, CD19, CD34, CD45, and HLA-DR antibodies (BD, USA) were added to the aliquots and incubated on ice for 30 min. After incubation, cells were washed twice, suspended with staining buffer and detected by flow cytometry immediately.

### Multi-differentiation of MSCs

To test the multi-differentiation ability of isolated cells, adipogenic, osteogenic and chondrogenic differentiation was induced, individually. All differentiation-induced media were prepared freshly. For adipogenic and osteogenic differentiation, MSCs at passage 2–6 were seeded in a six-well plate (3×10^5^/well) and cultured overnight. On the next day, the cells were washed with PBS twice and induction medium (Cyagen, China) once firstly, and then maintained in induction medium for adipogenic or osteogenic differentiation. The medium was changed every 3 days for 3 weeks. Then, the cells were stained with oil red O for adipogenicity and alizarin red S for osteogenesis, respectively, and observed under an inverted phase contrast microscope (Olympus, Japan). For chondrogenic differentiation, 2 × 10^6^ MSCs at passage 4–6 were harvested in a 15 mL centrifuge tube and washed with PBS twice and induction medium (Cyagen, China) once. After the last wash, supernatant was sucked carefully and thoroughly. 1 mL induction medium were added gently along the pipe wall to avoid disturbing the pellet. The tube with a loosened cap on it was placed in the cell culture incubator, which was humidified at 37° C with 5 % CO_2_. Medium was changed every 3 days by using a pipet. After 28 days, the chondrocyte pellet was fixed with neutral buffered formalin, embedded in paraffin, cut into 5-μm sections onto slides, stained with 1 % toluidine blue/1 % sodium borate, and observed under an inverted phase contrast microscope.

### Production of lentivirus

To label tumor cells and MSCs in co-culture system *in vitro* or animal study *in vivo*, we constructed lentiviral expression vectors encoding label genes firstly. The firefly luciferase (fLuc) sequence which had been amplified from pGL3 basic plasmid (Promega, USA) was cloned into the lentiviral expression vector (System Biosciences, SBI, USA) with a tag gene CopGFP. The DsRed sequence which had been amplified from plasmid DsRed-rab9 WT (Addgene, USA) was cloned into the lentiviral expression vector in place of the tag gene CopGFP. Secondly, the lentiviral particles were produced by transient transfection of 293T cells according to SBI’s protocol. The supernatant of cell culture medium was collected after 48 hours of post-transfection. The lentivirus containing medium was spun at 500g for 5 min, filtered through a 0.45 μm pore size filter (Millipore), and used to transduce tumor cells or MSCs immediately or stored at −80° C.

### Transduction of lentiviral particles

A549, H1299 and MSCs were plated at a density of 2×10^5^ per well in T-25cm^2^ cell culture flasks and incubated overnight at 37° C, individually. On the next day, culture medium was removed and 3 ml of fresh medium containing the corresponding lentiviral supernatant at MOI 8 and 8 mg/ml of polybrene (Sigma, USA) was added. And then the flasks were centrifuged at the speed of 1000 rpm at 25° C for 1.5 hours before taken back into the cell incubator. The medium was replaced by culture medium 8 hours later. Cells were incubated for the indicated time, and targeted gene expression (luciferase and CopGFP fluorescence for A549 and H1299; DsRed fluorescence for MSCs) was evaluated by luciferase assay (Promega, USA) or fluorescence microscope (Nikon, Japan).

### Transwell assay

To investigate the influence of LC-MSCs on invasion of tumor cells, we co-cultured tumor cells and LC-MSCs directly *in vitro*. Briefly, the transwell inserts were coated with matrigel (BD, USA), put in 24-well tissue culture plate, and then incubated in 5% CO_2_ incubator at 37° C for 5 hours. Excess matrigel that did not freeze was aspired before cell plating. A549 and H1299 cells that stably expressed CopGFP fluorescence and LC-MSCs were collected, washed twice with PBS and suspended in serum-free medium, respectively. Adjust the density of cells with serum-free medium. Add 20% FSC medium into the lower base of inserts. Then tumor cells and LC-MSCs were mixed well in 150μL serum-free medium (2.5×10^4^ cells for each; or 5×10^3^ tumor cells and 5×10^4^ MSCs) and plated in the upper base of the inserts. Incubate cultures for 24 hours in 5% CO_2_ incubator at 37° C. At the end of culture, erase the matrigel on the upper face pf the transwell membrane, and then count the number of tumor cells that migrated through the transwell membrane in five random fields under fluorescence microscope. Tumor cells plated alone were used as control. TF-MSCs were also co-incubated with tumor cells to compare the different effect between tumor and tumor-free derived MSCs. Experiments were repeated in triplicate. We also added the supernatants of LC/TF-MSCs in the culture media of tumor cells in transwell assay to evaluate the changes of invasion ability of tumor cells.

Additionally, we used transwell assay to the tropism of tumor cells induced by LC-MSCs. Briefly, 10^6^, 5×10^5^ and 10^5^ LC-MSCs in 2mL serum-free medium were incubated in 5% CO2 incubator at 37° C for 48 hours respectively. Then the culture medium from LC-MSCs was used as conditioned medium. Serum-free culture medium was used as control. Add the conditioned medium into the lower base of transwell inserts. 2×10^4^ tumor cells that was suspended in serum-free culture medium containing 0.2% BSA were plated in the upper base of transwell inserts. Put the transwell system in cell incubator for 5 hours. Then take out of the inserts. Slightly remove the cells that did not migrate through transwell membrane. Then stain the cells on the lower side of transwell membrane with 0.1% crystal violet. The number of cells that had migrated to the lower side of the membrane was counted under a light microscope with five high-power fields. Experiments were done in triplicate.

### Wound healing assay

To test tumor cell migration *in vitro*, we used wound healing assay in co-culture condition. Firstly, A549 and H1299 cells labeled with CopGFP fluorescence and LC-MSCs were collected, respectively. Then tumor cells and LC-MSCs at the ratio of 1:1 (2.5×10^5^ for each) or 1:10 (5×10^4^ tumor cells and 5×10^5^ LC-MSCs) were mixed well in 2 mL 10% FSC culture medium and incubated in 6-well tissue culture plate in 5% CO2 incubator at 37° C overnight. On the second day, cells should reach 80% confluence as monolayer. Gently and slowly scratch the monolayer with a new 1 mL pipette tip across the center of the well. Scratch another two straight lines parallel to the first one. Gently wash the wells twice with medium to remove the detached cells. Replenish the well with fresh medium. Take photos for the scratched monolayer for the first time. Grow the cells for additional 48 hours. Take photos for the scratched monolayer for the second time. The configurations of the microscope should be the same as the first time. The migrated ratio was calculated as (the first distance - the second distance)/ the first distance×100%.

### Quantitative real-time PCR

To evaluate the EMT and stem-like associated gene expression in transcriptional levels, we applied real-time PCR in the study. Total RNA was extracted from tumor cells with TRIzol (Gibco, USA), and cDNA was synthesized using PrimeScript™ RT Master Mix (TaKaRa, Japan) according to the manufacturer’s instructions. Real-time PCR amplification was performed with the 7500 real time PCR system (Applied Biosystems, USA) using SYBR Green qPCR kit (TaKaRa, Japan). The PCR conditions included 40 cycles of 95° C for 5 s and 60° C for 34 s. The primer pairs used for real-time PCR were described in the supplementary data (Supplementary Table 2). The relative expression levels of the target gene were evaluated by the 2^−ΔΔCT^ method, and β-actin gene expression was used for normalization of each sample.

### Western blotting

To evaluate the expression of EMT associated gene expression in protein levels, we used western blot in the study. Briefly, cells were lysed and protein was extracted using M-PER (Pierce, Rockford, IL, USA) plus protease inhibitor cocktail (Halt; Pierce). Ultrasonic the cell lysis in ice bath (300W for 4 seconds and rest for 8 seconds. Repeat three times.) was Protein concentrations were determined using BCA assay (Pierce). Aliquots of protein lysates were separated on SDS−polyacrylamide gels and transferred onto PVDF membrane, which was blocked with 5% blotting grade milk (Bio-Rad, USA) in PBST (0.1% Tween 20 in PBS). The membrane was then hybridized with the indicated primary antibodies to human E-cadherin, N-cadherin, β-catenin, vimentin, snail, slug and β-actin (Cell Signaling Technology, USA) followed by corresponding secondary antibodies conjugated with horseradish peroxidase, and then detected using a chemiluminescence assay (Millipore, USA). Membranes were exposed to X-ray film to visualize the bands. The band of β-actin was used as endogenous control.

### Spheroid culture

To investigate the stem-like characteristics of tumor cells induced by LC-MSCs or TF-MSCs, we tested the ability of spheroid formation of co-cultured cells by using 3D cell culture system. Briefly, 10^4^ A549 cells that had been labeled with CopGFP florescence and MSCs that had been labeled with DsRed florescence were co-plated at the cell ratio of 1:1, 1:3 (3×10^3^ MSCs), or 1: 10 (10^3^ MSCs) in six repeated wells of 96-well black round bottom polystyrene ultra-low attachment microplates (Corning, USA) in a volume of 0.2 mL. Centrifuge the microplates at the speed of 800 rpm for 5min to spin down cells in the bottom of the wells. Observe the sphere formation under an inverted fluorescence microscope at indicated time.

### *In vivo* experiments

To test the effect of MSCs on tumor progression *in vivo*, animal studies in two mouse models were carried out. Animal research was performed in compliance with the Guide for the Care and Use of Animal Ethics Committee of Tianjin Medical University (Tianjin, PR China). Female Balb/c nude mice aged 5−6 weeks (Beijing Vital River Laboratory Animal Technology Co., Ltd.; Beijing, China) were raised under pathogen-free conditions with irradiated fodder. A459 cells were firstly labeled with firefly luciferase (A459.Lu) by lentivirus transduction. In the subcutaneous mouse model, 10^6^, 2×10^4^ or 10^3^ A459.Lu cells respectively with or without MSCs (10^6^ or 2×10^5^) were implanted subcutaneously into the right flank of each mouse in a volume of 0.2 mL using 1 mL syringe with 18g 1 1/2” needle tip (BD, USA). In lung cancer metastasis mouse model, 5×10^5^ A459.Lu cells with or without 5×10^5^ MSCs were injected intravenously into the tail vein of each mouse in a volume of 0.1 mL using 1mL syringe with 28G 1/2” needle tip (BD, USA). The growth of tumor was monitored by bioluminescence imaging (BLI) (*In Vivo* Imaging System-Xenogen 100 system; Caliper Lifesciences, USA). For BLI, mice were administrated intraperitoneally D-luciferin (15 mg/mL in PBS, Promega, USA) at a dosage of 150 mg/kg 10 min prior to imaging. At the end of experiment, tumor and sentinel lymph nodes were taken out from the sacrificed mice. Histopathological test was used to confirm the tumor tissues. Immunohistochemistry test was used to the expression of Ki67 to evaluate the proliferation of tumor cells.

### Statistical analyses

Experimental data are presented as the mean and standard deviation (SD) values. Statistical analysis was performed using SPSS22.0 software. Differences between groups were examined for significant differences by ANOVA LSD or Dunnett post hoc procedure. Limiting dilution analyses were performed based on previous report [49], using the limdil function of the “statmod” package (http://bioinf.wehi.edu.au/software/elda/index.html). Lung cancer-initiating cell frequencies were compared using likelihood ratio tests. Values of P < 0.05 were considered to be statistically significant. Data of each patient was assessed in triplicate.

## Supplementary Material

Supplementary Figures

Supplementary Tables

## References

[1] Marofi F, Vahedi G, Hasanzadeh A, Salarinasab S, Arzhanga P, Khademi B, Farshdousti Hagh M. Mesenchymal stem cells as the game-changing tools in the treatment of various organs disorders: mirage or reality? J Cell Physiol. 2019; 234:1268–88. 10.1002/jcp.27152 30191962

[2] Rodriguez AM, Nakhle J, Griessinger E, Vignais ML. Intercellular mitochondria trafficking highlighting the dual role of mesenchymal stem cells as both sensors and rescuers of tissue injury. Cell Cycle. 2018; 17:712–21. 10.1080/15384101.2018.1445906 29582715 PMC5969546

[3] Wang Z, Sun D. Adipose-derived mesenchymal stem cells: a new tool for the treatment of renal fibrosis. Stem Cells Dev. 2018; 27:1406–11. 10.1089/scd.2017.0304 30032706

[4] Krueger TE, Thorek DL, Denmeade SR, Isaacs JT, Brennen WN. Concise review: mesenchymal stem cell-based drug delivery: the good, the bad, the ugly, and the promise. Stem Cells Transl Med. 2018; 7:651–63. 10.1002/sctm.18-0024 30070053 PMC6127224

[5] Pavon LF, Sibov TT, de Souza AV, da Cruz EF, Malheiros SM, Cabral FR, de Souza JG, Boufleur P, de Oliveira DM, de Toledo SR, Marti LC, Malheiros JM, Paiva FF, et al. Tropism of mesenchymal stem cell toward CD133^+^ stem cell of glioblastoma *in vitro* and promote tumor proliferation *in vivo*. Stem Cell Res Ther. 2018; 9:310. 10.1186/s13287-018-1049-0 30413179 PMC6234773

[6] Kidd S, Spaeth E, Dembinski JL, Dietrich M, Watson K, Klopp A, Battula VL, Weil M, Andreeff M, Marini FC. Direct evidence of mesenchymal stem cell tropism for tumor and wounding microenvironments using *in vivo* bioluminescent imaging. Stem Cells. 2009; 27:2614–23. 10.1002/stem.187 19650040 PMC4160730

[7] Karnoub AE, Dash AB, Vo AP, Sullivan A, Brooks MW, Bell GW, Richardson AL, Polyak K, Tubo R, Weinberg RA. Mesenchymal stem cells within tumour stroma promote breast cancer metastasis. Nature. 2007; 449:557–63. 10.1038/nature06188 17914389

[8] Mi Z, Bhattacharya SD, Kim VM, Guo H, Talbot LJ, Kuo PC. Osteopontin promotes CCL5-mesenchymal stromal cell-mediated breast cancer metastasis. Carcinogenesis. 2011; 32:477–87. 10.1093/carcin/bgr009 21252118 PMC3105582

[9] Correa D, Somoza RA, Lin P, Schiemann WP, Caplan AI. Mesenchymal stem cells regulate melanoma cancer cells extravasation to bone and liver at their perivascular niche. Int J Cancer. 2016; 138:417–27. 10.1002/ijc.29709 26235173 PMC4882929

[10] de Souza LE, Malta TM, Kashima Haddad S, Covas DT. Mesenchymal stem cells and pericytes: to what extent are they related? Stem Cells Dev. 2016; 25:1843–52. 10.1089/scd.2016.0109 27702398

[11] Guilloton F, Caron G, Ménard C, Pangault C, Amé-Thomas P, Dulong J, De Vos J, Rossille D, Henry C, Lamy T, Fouquet O, Fest T, Tarte K. Mesenchymal stromal cells orchestrate follicular lymphoma cell niche through the CCL2-dependent recruitment and polarization of monocytes. Blood. 2012; 119:2556–67. 10.1182/blood-2011-08-370908 22289889

[12] Yan X, Zhang D, Wu W, Wu S, Qian J, Hao Y, Yan F, Zhu P, Wu J, Huang G, Huang Y, Luo J, Liu X, et al. Mesenchymal stem cells promote hepatocarcinogenesis via lncRNA-MUF interaction with ANXA2 and miR-34a. Cancer Res. 2017; 77:6704–16. 10.1158/0008-5472.CAN-17-1915 28947421

[13] Yu PF, Huang Y, Xu CL, Lin LY, Han YY, Sun WH, Hu GH, Rabson AB, Wang Y, Shi YF. Downregulation of CXCL12 in mesenchymal stromal cells by TGFβ promotes breast cancer metastasis. Oncogene. 2017; 36:840–49. 10.1038/onc.2016.252 27669436 PMC5311419

[14] Gonzalez DM, Medici D. Signaling mechanisms of the epithelial-mesenchymal transition. Sci Signal. 2014; 7:re8. 10.1126/scisignal.2005189 25249658 PMC4372086

[15] Lamouille S, Xu J, Derynck R. Molecular mechanisms of epithelial-mesenchymal transition. Nat Rev Mol Cell Biol. 2014; 15:178–96. 10.1038/nrm3758 24556840 PMC4240281

[16] Dongre A, Weinberg RA. New insights into the mechanisms of epithelial-mesenchymal transition and implications for cancer. Nat Rev Mol Cell Biol. 2019; 20:69–84. 10.1038/s41580-018-0080-4 30459476

[17] Wu MJ, Chen YS, Kim MR, Chang CC, Gampala S, Zhang Y, Wang Y, Chang CY, Yang JY, Chang CJ. Epithelial-mesenchymal transition directs stem cell polarity via regulation of mitofusin. Cell Metab. 2019; 29:993–1002.e6. 10.1016/j.cmet.2018.11.004 30527740

[18] Jung HY, Fattet L, Yang J. Molecular pathways: linking tumor microenvironment to epithelial-mesenchymal transition in metastasis. Clin Cancer Res. 2015; 21:962–68. 10.1158/1078-0432.CCR-13-3173 25107915 PMC4320988

[19] Li H, Xu F, Li S, Zhong A, Meng X, Lai M. The tumor microenvironment: an irreplaceable element of tumor budding and epithelial-mesenchymal transition-mediated cancer metastasis. Cell Adh Migr. 2016; 10:434–46. 10.1080/19336918.2015.1129481 26743180 PMC4986710

[20] Costea DE, Hills A, Osman AH, Thurlow J, Kalna G, Huang X, Pena Murillo C, Parajuli H, Suliman S, Kulasekara KK, Johannessen AC, Partridge M. Identification of two distinct carcinoma-associated fibroblast subtypes with differential tumor-promoting abilities in oral squamous cell carcinoma. Cancer Res. 2013; 73:3888–901. 10.1158/0008-5472.CAN-12-4150 23598279

[21] Yu Y, Xiao CH, Tan LD, Wang QS, Li XQ, Feng YM. Cancer-associated fibroblasts induce epithelial-mesenchymal transition of breast cancer cells through paracrine TGF-β signalling. Br J Cancer. 2014; 110:724–32. 10.1038/bjc.2013.768 24335925 PMC3915130

[22] Shintani Y, Fujiwara A, Kimura T, Kawamura T, Funaki S, Minami M, Okumura M. IL-6 secreted from cancer-associated fibroblasts mediates chemoresistance in NSCLC by increasing epithelial-mesenchymal transition signaling. J Thorac Oncol. 2016; 11:1482–92. 10.1016/j.jtho.2016.05.025 27287412

[23] Zhao L, Ji G, Le X, Luo Z, Wang C, Feng M, Xu L, Zhang Y, Lau WB, Lau B, Yang Y, Lei L, Yang H, et al. An integrated analysis identifies STAT4 as a key regulator of ovarian cancer metastasis. Oncogene. 2017; 36:3384–96. 10.1038/onc.2016.487 28114283

[24] Anderson P, Carrillo-Gálvez AB, García-Pérez A, Cobo M, Martín F. CD105 (endoglin)-negative murine mesenchymal stromal cells define a new multipotent subpopulation with distinct differentiation and immunomodulatory capacities. PLoS One. 2013; 8:e76979. 10.1371/journal.pone.0076979 24124603 PMC3790740

[25] Han B, Zhou L, Guan Q, da Roza G, Wang H, Du C. *In vitro* expansion and characterization of mesenchymal stromal cells from peritoneal dialysis effluent in a human protein medium. Stem Cells Int. 2018; 2018:5868745. 10.1155/2018/5868745 30402111 PMC6192083

[26] Salvolini E, Orciani M, Vignini A, Mattioli-Belmonte M, Mazzanti L, Di Primio R. Skin-derived mesenchymal stem cells (S-MSCs) induce endothelial cell activation by paracrine mechanisms. Exp Dermatol. 2010; 19:848–50. 10.1111/j.1600-0625.2010.01104.x 20629738

[27] Cleary MA, Narcisi R, Focke K, van der Linden R, Brama PA, van Osch GJ. Expression of CD105 on expanded mesenchymal stem cells does not predict their chondrogenic potential. Osteoarthritis Cartilage. 2016; 24:868–72. 10.1016/j.joca.2015.11.018 26687821

[28] Chaturvedi P, Gilkes DM, Takano N, Semenza GL. Hypoxia-inducible factor-dependent signaling between triple-negative breast cancer cells and mesenchymal stem cells promotes macrophage recruitment. Proc Natl Acad Sci USA. 2014; 111:E2120–29. 10.1073/pnas.1406655111 24799675 PMC4034192

[29] Shi Y, Du L, Lin L, Wang Y. Tumour-associated mesenchymal stem/stromal cells: emerging therapeutic targets. Nat Rev Drug Discov. 2017; 16:35–52. 10.1038/nrd.2016.193 27811929

[30] Yan C, Song X, Yu W, Wei F, Li H, Lv M, Zhang X, Ren X. Human umbilical cord mesenchymal stem cells delivering sTRAIL home to lung cancer mediated by MCP-1/CCR2 axis and exhibit antitumor effects. Tumour Biol. 2016; 37:8425–35. 10.1007/s13277-015-4746-7 26733169

[31] Yan C, Yang M, Li Z, Li S, Hu X, Fan D, Zhang Y, Wang J, Xiong D. Suppression of orthotopically implanted hepatocarcinoma in mice by umbilical cord-derived mesenchymal stem cells with sTRAIL gene expression driven by AFP promoter. Biomaterials. 2014; 35:3035–43. 10.1016/j.biomaterials.2013.12.037 24406219

[32] De Boeck A, Pauwels P, Hensen K, Rummens JL, Westbroek W, Hendrix A, Maynard D, Denys H, Lambein K, Braems G, Gespach C, Bracke M, De Wever O. Bone marrow-derived mesenchymal stem cells promote colorectal cancer progression through paracrine neuregulin 1/HER3 signalling. Gut. 2013; 62:550–60. 10.1136/gutjnl-2011-301393 22535374

[33] Roccaro AM, Sacco A, Maiso P, Azab AK, Tai YT, Reagan M, Azab F, Flores LM, Campigotto F, Weller E, Anderson KC, Scadden DT, Ghobrial IM. BM mesenchymal stromal cell-derived exosomes facilitate multiple myeloma progression. J Clin Invest. 2013; 123:1542–55. 10.1172/JCI66517 23454749 PMC3613927

[34] Beckermann BM, Kallifatidis G, Groth A, Frommhold D, Apel A, Mattern J, Salnikov AV, Moldenhauer G, Wagner W, Diehlmann A, Saffrich R, Schubert M, Ho AD, et al. VEGF expression by mesenchymal stem cells contributes to angiogenesis in pancreatic carcinoma. Br J Cancer. 2008; 99:622–31. 10.1038/sj.bjc.6604508 18665180 PMC2527820

[35] Huang WH, Chang MC, Tsai KS, Hung MC, Chen HL, Hung SC. Mesenchymal stem cells promote growth and angiogenesis of tumors in mice. Oncogene. 2013; 32:4343–54. 10.1038/onc.2012.458 23085755

[36] Bexell D, Gunnarsson S, Tormin A, Darabi A, Gisselsson D, Roybon L, Scheding S, Bengzon J. Bone marrow multipotent mesenchymal stroma cells act as pericyte-like migratory vehicles in experimental gliomas. Mol Ther. 2009; 17:183–90. 10.1038/mt.2008.229 18985030 PMC2834971

[37] Crisan M, Yap S, Casteilla L, Chen CW, Corselli M, Park TS, Andriolo G, Sun B, Zheng B, Zhang L, Norotte C, Teng PN, Traas J, et al. A perivascular origin for mesenchymal stem cells in multiple human organs. Cell Stem Cell. 2008; 3:301–13. 10.1016/j.stem.2008.07.003 18786417

[38] Ye X, Tam WL, Shibue T, Kaygusuz Y, Reinhardt F, Ng Eaton E, Weinberg RA. Distinct EMT programs control normal mammary stem cells and tumour-initiating cells. Nature. 2015; 525:256–60. 10.1038/nature14897 26331542 PMC4764075

[39] Guo W, Keckesova Z, Donaher JL, Shibue T, Tischler V, Reinhardt F, Itzkovitz S, Noske A, Zürrer-Härdi U, Bell G, Tam WL, Mani SA, van Oudenaarden A, Weinberg RA. Slug and Sox9 cooperatively determine the mammary stem cell state. Cell. 2012; 148:1015–28. 10.1016/j.cell.2012.02.008 22385965 PMC3305806

[40] Mishra PJ, Mishra PJ, Humeniuk R, Medina DJ, Alexe G, Mesirov JP, Ganesan S, Glod JW, Banerjee D. Carcinoma-associated fibroblast-like differentiation of human mesenchymal stem cells. Cancer Res. 2008; 68:4331–39. 10.1158/0008-5472.CAN-08-0943 18519693 PMC2725025

[41] Shinagawa K, Kitadai Y, Tanaka M, Sumida T, Onoyama M, Ohnishi M, Ohara E, Higashi Y, Tanaka S, Yasui W, Chayama K. Stroma-directed imatinib therapy impairs the tumor-promoting effect of bone marrow-derived mesenchymal stem cells in an orthotopic transplantation model of colon cancer. Int J Cancer. 2013; 132:813–23. 10.1002/ijc.27735 22821812

[42] Chen Y, LeBleu VS, Carstens JL, Sugimoto H, Zheng X, Malasi S, Saur D, Kalluri R. Dual reporter genetic mouse models of pancreatic cancer identify an epithelial-to-mesenchymal transition-independent metastasis program. EMBO Mol Med. 2018; 10:e9085. 10.15252/emmm.201809085 30120146 PMC6180301

[43] Puram SV, Tirosh I, Parikh AS, Patel AP, Yizhak K, Gillespie S, Rodman C, Luo CL, Mroz EA, Emerick KS, Deschler DG, Varvares MA, Mylvaganam R, et al. Single-cell transcriptomic analysis of primary and metastatic tumor ecosystems in head and neck cancer. Cell. 2017; 171:1611–24.e24. 10.1016/j.cell.2017.10.044 29198524 PMC5878932

[44] McFaline-Figueroa JL, Hill AJ, Qiu X, Jackson D, Shendure J, Trapnell C. A pooled single-cell genetic screen identifies regulatory checkpoints in the continuum of the epithelial-to-mesenchymal transition. Nat Genet. 2019; 51:1389–98. 10.1038/s41588-019-0489-5 31477929 PMC6756480

[45] Bierie B, Pierce SE, Kroeger C, Stover DG, Pattabiraman DR, Thiru P, Liu Donaher J, Reinhardt F, Chaffer CL, Keckesova Z, Weinberg RA. Integrin-β4 identifies cancer stem cell-enriched populations of partially mesenchymal carcinoma cells. Proc Natl Acad Sci USA. 2017; 114:E2337–46. 10.1073/pnas.1618298114 28270621 PMC5373369

[46] Chaffer CL, San Juan BP, Lim E, Weinberg RA. EMT, cell plasticity and metastasis. Cancer Metastasis Rev. 2016; 35:645–54. 10.1007/s10555-016-9648-7 27878502

[47] Thiery JP, Lim CT. Tumor dissemination: an EMT affair. Cancer Cell. 2013; 23:272–73. 10.1016/j.ccr.2013.03.004 23518345

[48] Von der Heide EK, Neumann M, Vosberg S, James AR, Schroeder MP, Ortiz-Tanchez J, Isaakidis K, Schlee C, Luther M, Jöhrens K, Anagnostopoulos I, Mochmann LH, Nowak D, et al. Molecular alterations in bone marrow mesenchymal stromal cells derived from acute myeloid leukemia patients. Leukemia. 2017; 31:1069–78. 10.1038/leu.2016.324 27833093

[49] Quintana E, Shackleton M, Sabel MS, Fullen DR, Johnson TM, Morrison SJ. Efficient tumour formation by single human melanoma cells. Nature. 2008; 456:593–98. 10.1038/nature07567 19052619 PMC2597380

