# Supplementary material for: Lung cancer-associated mesenchymal stem cells promote tumor metastasis and tumorigenesis by induction of epithelial–mesenchymal transition and stem-like reprogram
Source: Aging (Albany NY). 2021 Mar 19;13(7):9780–800. doi: 10.18632/aging.202732 (PMC8064219; doi:10.18632/aging.202732)
Supplement: Supplementary Figures [file aging-13-202732-s001.pdf]

SUPPLEMENTARY FIGURES

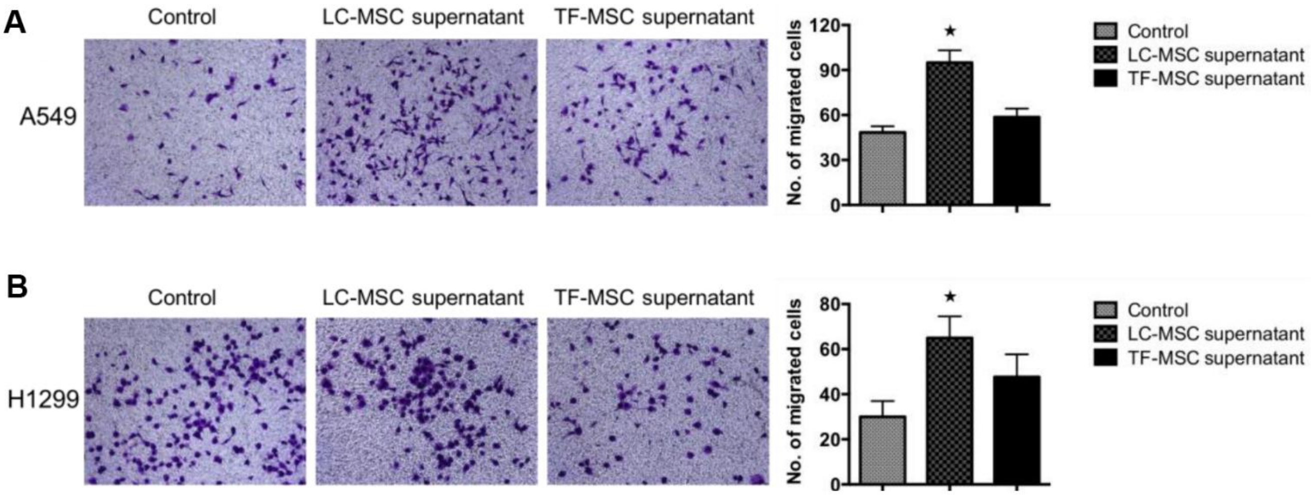

Supplementary Figure 1. Induced invasion by the supernatant from LC-MSCs. The conditional culture media from LC-MSCs or TF-MSCs were collected after 48 hours of incubation. The conditional media were mixed with RPMI 1640 at the ratio of 1:1. A549 (**A**) and H1299 (**B**) cells were then resuspended in the mixture culture media and added into the above base of the Transwell insert where matrigel had been plated. Cells were incubated for 48 hours. The cells that migrated across the matrigel and membrane were counted. *, P < 0.05 were considered to be statistically significant.

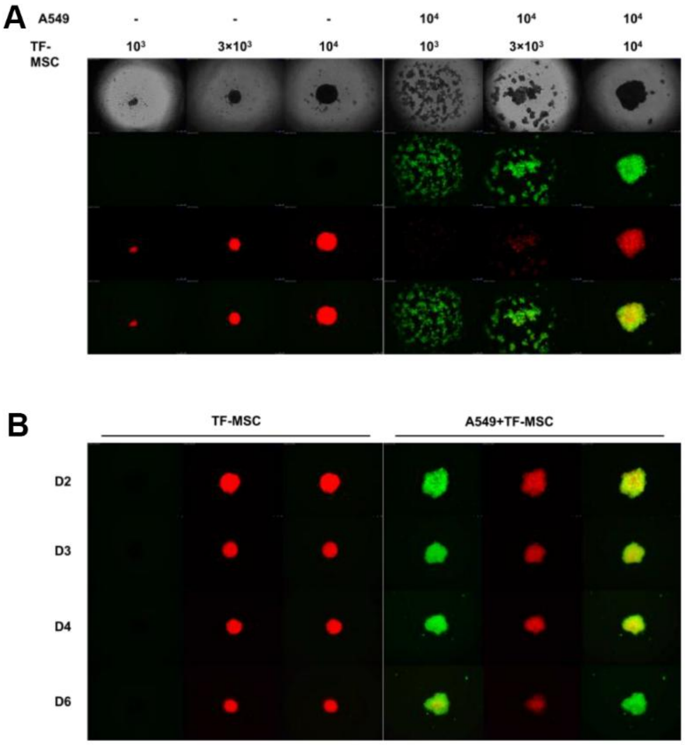

Supplementary Figure 2. Spheroid formation by TF-MSCs. (**A**) Different cell ratio of A549.CopGFP and TF-MSCs.DsRed were co-incubated in 96-well 3D cell culture plates. The spheroid formation was observed under inverted fluorescence microscope on the second day of plating cells. (**B**) Spheroid grew observed at the indicated time.

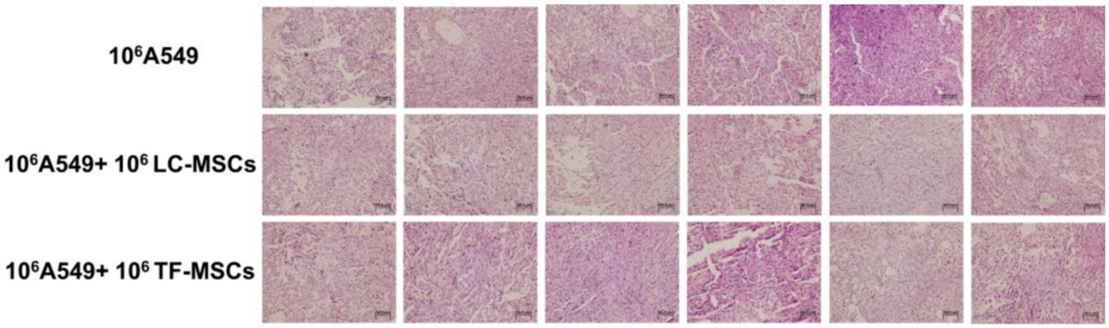

Supplementary Figure 3. H.E staining for tumor tissues. 10^6 each of A549. Luc cells and LC-MSCs or TF-MSCs were co-implanted subcutaneously into female Balb/c nude mice. Histopathological test for tumors was carried out on day 42 at the end of experiment. The tumor cells in the co-implanted groups showed more derangement distribution which was disordered by fibroblast-like cells than that in A549 group.

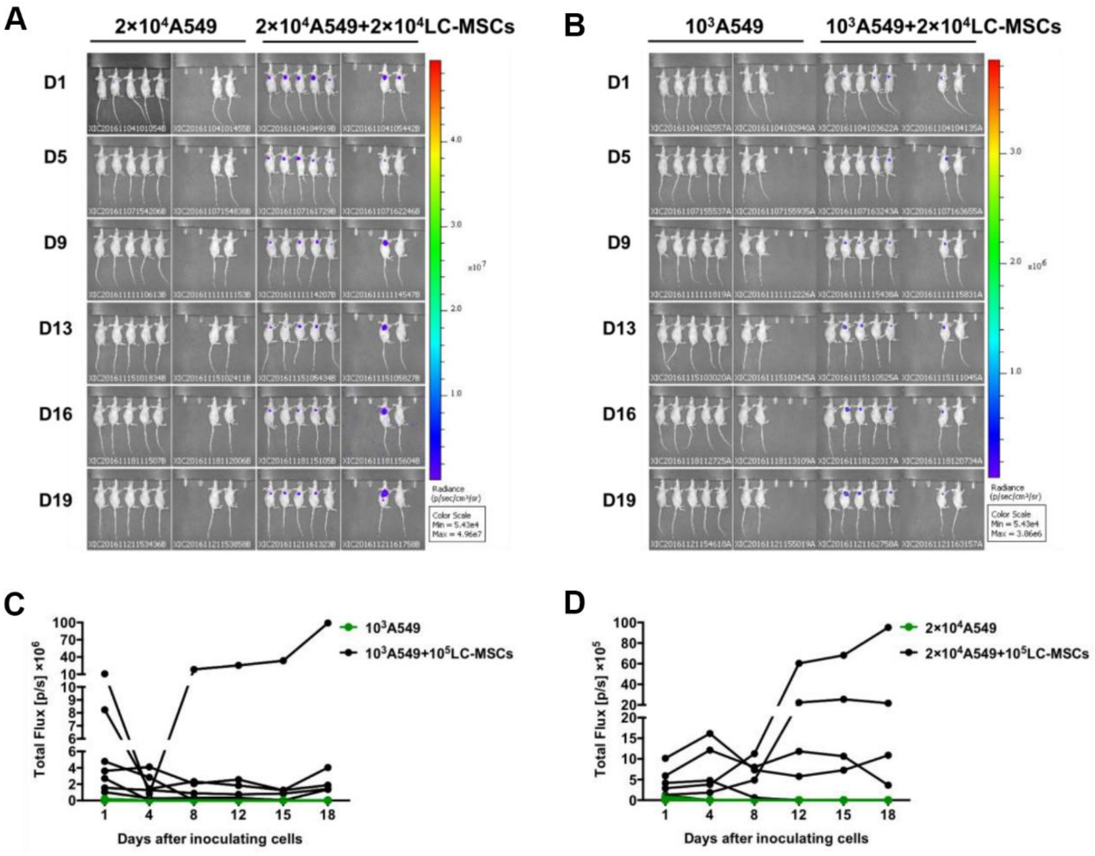

Supplementary Figure 4. Increased tumorigenesis by LC-MSCs. Low number of A549.Luc cells and LC-MSCs were co-implanted subcutaneously into female Balb/c nude mice. (**A**, **C**) Bioluminescence images at the indicated time. (**B**, **D**) Total bioluminescence of tumors at the indicated time.

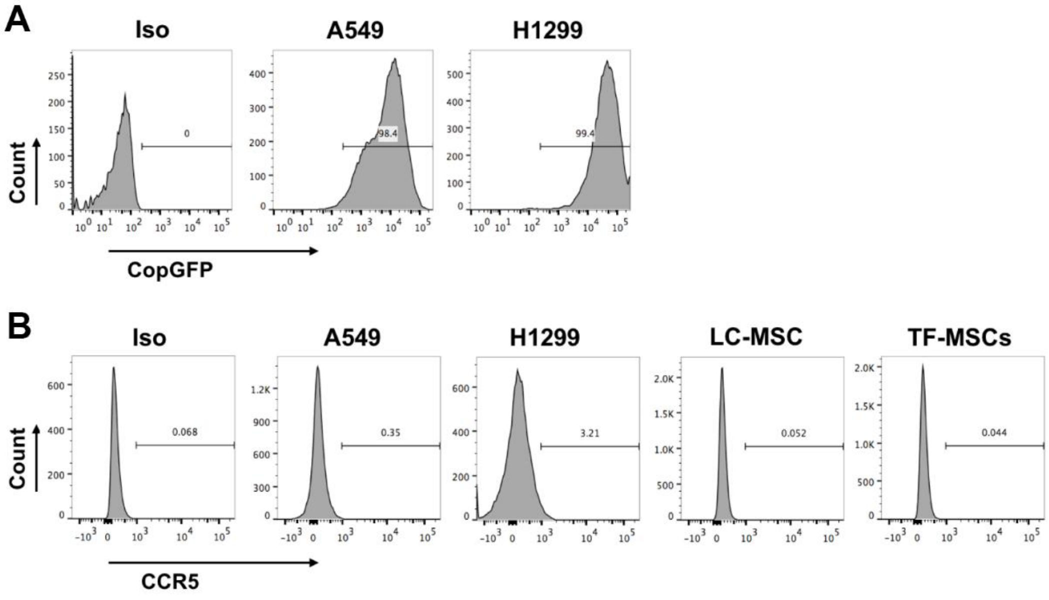

Supplementary Figure 5. Cytometry flow test for gene expression. (**A**) A549 and H1299 cells stably expressed CopGFP fluorescence by transduction of lentiviral particles encoding CopGFP gene sequence. (**B**) CCR5 expression on tumor cells and MSCs. LC-MSC, lung cancer-associated MSC. TF-MSC, adjacent tumor-free tissue derived MSC.

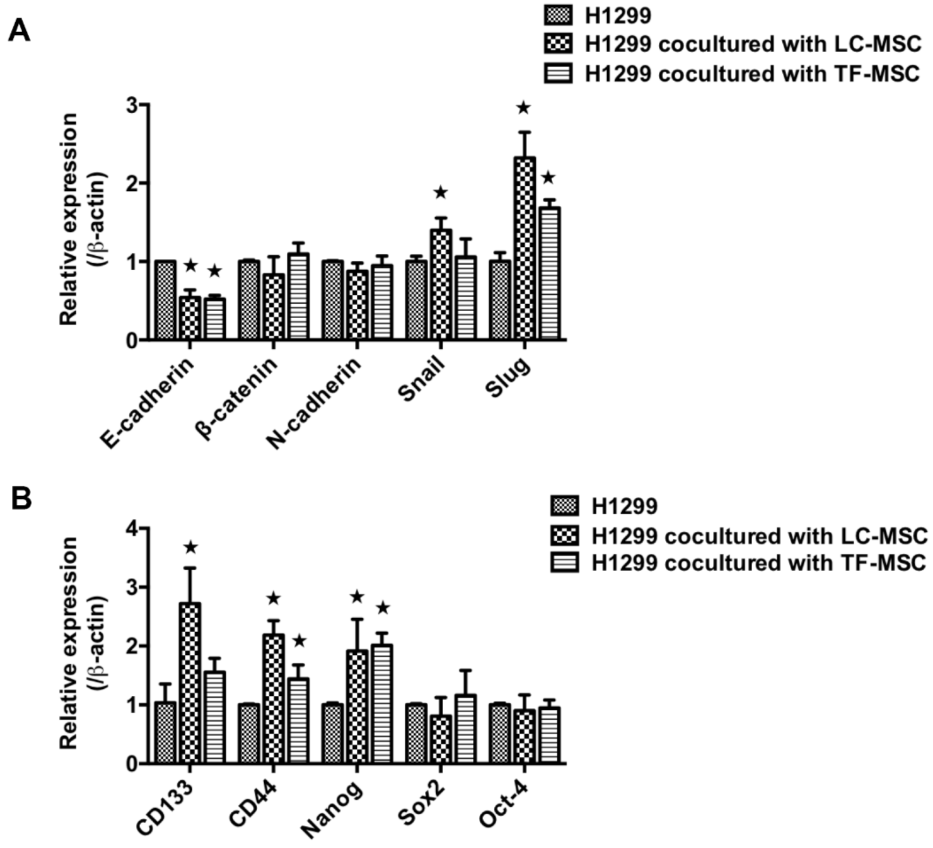

Supplementary Figure 6. EMT and stem-like characteristics induced by LC-MSCs. H1299 cells were treated with the supernatants from LC-MSCs or TF-MSCs for 48 hours. The expression of EMT gene (**A**) and stem-like gene (**B**) were tested by realtime PCR assay. *, $P < 0.05$ were considered to be statistically significant.
