# Supplementary material for: Lung cancer-associated mesenchymal stem cells promote tumor metastasis and tumorigenesis by induction of epithelial–mesenchymal transition and stem-like reprogram
Source: Aging (Albany NY). 2021 Mar 19;13(7):9780–800. doi: 10.18632/aging.202732 (PMC8064219; doi:10.18632/aging.202732)
Supplement: Supplementary Tables [file aging-13-202732-s002.pdf]

SUPPLEMENTARY TABLES

Supplementary Table 1. Patient baseline characteristics.

Patient ID	Gender	Age	Smoke	Drink	Location	Pathologic stage	Pathology	EGFR status	ALK status	Treatment
1	Female	60	no	no	Right, low	T1N0M0	Squamous cell carcinoma	no	no	surgery
2	Male	79	yes	no	Right, up	T2N0M0	adenocarcinoma	No	no	Surgery
3	Male	42	yes	yes	Right, up	T2N3M0	adenocarcinoma	no	yes	Surgery+chemo
4	Male	61	no	yes	Left, up	T2N0M0	Squamous cell carcinoma	yes	no	Surgery+chemo
5	Female	63	yes	no	Right, up	T3N2M0	adenocarcinoma	yes	no	Surgery+chemo+rad

Supplementary Table 2. Primers for quantitative real-time PCR.

Gene	Primers	
E-cadherin	5’ → 3’	TGCCCAGAAAATGAAAAAGG
	5’ → 3’	GTGTATGTGGCAATGCGTTC
N-cadherin	5’ → 3’	ACAGTGGCCACCTACAAAGG
	5’ → 3’	CCGAGATGGGGTTGATAATG
β-catenin	5’ → 3’	TGGTGACAGGGAAGACATCA
	5’ → 3’	CCATAGTGAAGGCGAACTGC
Snail	5’ → 3’	AGCCTGGGTGCCCTCAAGAT
	5’ → 3’	AGGTTGGAGCGGTCAGCGAA
Slug	5’ → 3’	TGCCTGTCATACCACAACCAGA
	5’ → 3’	GGAGGAGGTGTCAGATGGAGGA
CD133	5’ → 3’	CACCATTGACTTCTTGGTGCTG
	5’ → 3’	TGCATGCCATTTCCAAGTGG
CD44	5’ → 3’	CCTGTTCTCTCCTGTGAAAG
	5’ → 3’	TTTGCCAATCTCTTTCATTT
Nanog	5’ → 3’	GCAGTTCCAGCCAAATTCTC
	5’ → 3’	TTCACACGTCTTCAGGTTGC
SOX2	5’ → 3’	TGCGAGCGCTGCACAT
	5’ → 3’	GCAGCGTGTACTTATCCTTCTTCA
OCT-4	5’ → 3’	GTATTCAGCCAAACGACCATC
	5’ → 3’	CTGGTTCGCTTTCTCTTTCG
